# The Binding Specificity of PAB1 with Poly(A) mRNA, Regulated by Its Structural Folding

**DOI:** 10.3390/biomedicines10112981

**Published:** 2022-11-19

**Authors:** Monikaben Padariya, Umesh Kalathiya

**Affiliations:** International Centre for Cancer Vaccine Science, University of Gdansk, ul. Kładki 24, 80-822 Gdansk, Poland

**Keywords:** poly(A) mRNA, PABPC1, transcripts, molecular dynamics, protein stability, binding affinity, hydrogen bonds, cancer mutations

## Abstract

The poly(A)-binding protein cytoplasmic 1 (PAB1 or PABPC1) protein is associated with the long poly(A) mRNA tails, inducing stability. Herein, we investigated the dynamics of the PABPC1 protein, along with tracing its mRNA binding specificity. During molecular dynamics simulations (MDS), the R176-Y408 amino acids (RRM3–4 domains; RNA recognition motifs) initiated a folded structure that resulted in the formation of different conformations. The RRM4 domain formed high-frequency intramolecular interactions, despite such induced flexibility. Residues D45, Y54, Y56, N58, Q88, and N100 formed long-lasting interactions, and specifically, aromatic residues (Y14, Y54, Y56, W86, and Y140) gained a unique binding pattern with the poly(A) mRNA. In addition, the poly(A) mRNA motif assembled a PABPC1-specific conformation, by inducing movement of the center three nucleotides to face towards RRM1–2 domains. The majority of the high-frequency cancer mutations in PAB1 reside within the RRM4 domain and amino acids engaging in high-frequency interactions with poly(A) mRNA were found to be preserved in different cancer types. Except for the G123C variant, other studied cancer-derived mutants hindered the stability of the protein. Molecular details from this study will provide a detailed understanding of the PABPC1 structure, which can be used to modulate the activity of this gene, resulting in production of mutant peptide or neoantigens in cancer.

## 1. Introduction

**Figure 1:**
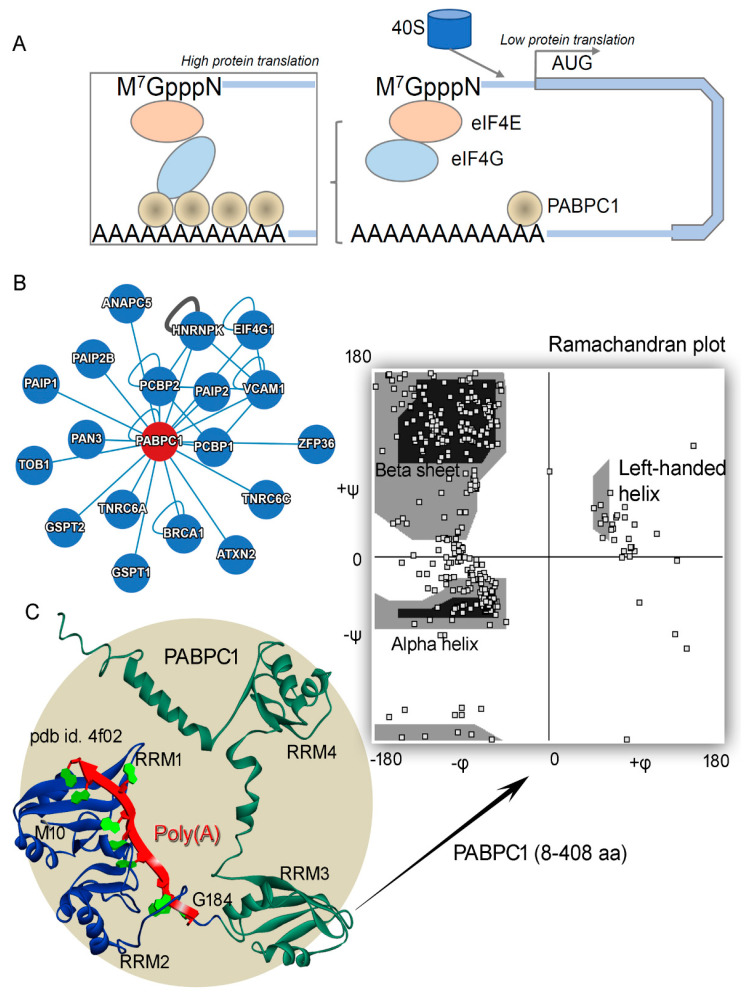
The poly(A)-binding protein cytoplasmic 1 (PAB1 or PABPC1) protein. (**A**) The conformational switch of eIF4G (eukaryotic translation initiation factor 4 G) gene depends on the PAB1 expression; i.e., in normal cells having low PAB1 expression, binding of eIF4G-PAB1 is lacking, whereas in cancer cells having high PAB1 expression, binding is induced between these proteins [9]. (**B**) The PAB1 protein network map retrieved from the Interactome database [10]. (**C**) Crystal structure of PAB1 (RRM1–2 domains; RNA recognition motifs) with the poly(A) mRNA (pdb id.: 4f02 [2] in blue; M10-G184 amino acids) superimposed with the modeled PAB1 tertiary structure (RRM1-4 domains; in green). The modeled PAB1 contains the following RNA recognition motifs domains: RRM1 (11–89 aa), RRM2 (99–175 aa), RRM3 (181–268 aa), and RRM4 (294–370 aa). The right panel represents the “Ramachandran plot” describing different secondary structures for the modeled PABPC1 structure, generated using the VMD (Visual Molecular Dynamics tool) [15].

The eukaryotic mRNAs with poly(A) tails bind to sequence-specific poly(A)-binding proteins, mediating synthesis of these tails [1]. One such protein is poly(A)-binding protein cytoplasmic 1 (PAB1 or PABPC1), associated with the long poly(A) mRNA tails inducing its stability. The PABPC1 protein, with a molecular weight of ~70 kDa, consists of four RNA recognition motifs (RRMs) linked through linker amino acids [1,2]. The “faux 3′-UTR” model explains that the proximity of the PABPC1 to the PTC (premature termination codons) is essential for NMD (nonsense-mediated mRNA decay) activation [2,3]. For poly(A) shortening, ribosome recruitment, and translation initiation, the PABPC protein plays a crucial role by binding directly to the poly(A) tail of mRNA in cytoplasm [1]. Along with high specific binding to poly(A) mRNA, the PABPC1 protein is found binding with lower affinity to poly(U) and poly(G) [4,5] Among different mechanisms of PABPC is the stimulation of initiation of translation by binding with the eIF4G protein (eukaryotic translation initiation factor 4 G), thus promoting the recruitment of 40S ribosome subunits similar as the eIF4E gene [6,7,8]. Such binding of PABPC1-eIF4G is found to be conserved in different species [1]. The conformational switch of the eIF4G gene depends on the PAB1 expression; i.e., in normal cells having low PAB1 expression, the binding of eIF4G-PAB1 is lacking (Figure 1), whereas in cancer cells with high PAB1 expression, binding is induced between these proteins [9,10].

Though the PABPC1 protein is proposed to be an antagonist of UPF1 (UP-frameshift 1) from the NMD pathway towards its mRNA activity, it has been found that deletion of either UPF1 or PAB1 significantly increases the production of novel peptide read-through [11,12,13]. Crucial roles of the poly(A)-binding protein for regulation in the translation termination have been investigated [11], and inefficient termination can result in the activation of the NMD process (initiating mRNA degradation). Different biochemical and structural studies have reported the binding patterns between the RNA recognition motifs with poly(A) mRNA, as well as interaction with different components [2,14]. However, detailed insights are still needed for the structural folding and dynamics of the full-length human PABPC1 protein. Due to significant correlation between mRNA degradation and stabilization, herein, we investigated the PABPC1 protein’s dynamics, along with tracing mRNA binding specificity (Figure 1A). Additionally, considering different cancer-derived mutations for the PABPC1 protein, we presented an overview of different hotspots [15], along with measuring the change in stability of the structure upon inserting point mutations. Monitoring the mRNA selectivity for PAB1, our findings suggest that aromatic amino acids (Y or W) are associated extensively with the poly(A) mRNA (Figure 1).

## 2. Materials and Methods

The crystal structure of PABPC1 containing the RRM1–2 domains with the poly(A) mRNA (pdb id: 4f02 [2] in blue; M10–G184 aa; Figure 1) was retrieved from the protein data bank (pdb; www.rcsb.org accessed on 3 May 2021). The modeled human PABPC1 structure containing all four RRMs was built using the SWISS-MODEL homology modeling approach [16,17]. For generating human PABPC1 (RRM1-4), the *Saccharomyces cerevisiae* PAB1 cryo-EM (cryogenic electron microscopy) structure from the 90A RNP-Pan2-Pan3 complex (pdb id.: 6r5k [14]) was considered as a template structure. Superimposing of crystal structure with the modeled PAB1 tertiary structure (Figure 1) was performed using the BIOVIA Discovery Studio (Dassault Systèmes, BIOVIA Corp., San Diego, CA, USA) pipeline. Different PABPC1 models in the presence or absence of the poly(A) mRNA were energy-minimized via applying CHARMM27 (Chemistry at Harvard Macromolecular Mechanics) forcefield in the Molecular Operating Environment (MOE; Chemical Computing Group Inc., Montreal, QC, Canada) package. Moreover, retrieving coordinates from the PAB1-simulated structure, the active sites were predicted using the “Alpha Shapes” construction geometric method implemented in the MOE modules (Chemical Computing Group Inc., Montreal, QC, Canada) [18]. The “Alpha Shapes” technique allowed us to construct the binding site of a protein, classified in the alpha sphere form as either “hydrophobic” or “hydrophilic (for lone pair active; LPA)”.

PABPC1 mutations retrieved from the cBioPortal database [19] were represented over the structure in the BIOVIA Discovery Studio (Dassault Systèmes, BIOVIA Corp., San Diego, CA, USA). Moreover, expression profiles of specific genes within a single dataset (type 1 diabetes (T1D) autoimmune disease), compared with the control samples (GSE60424, Neutrophils; GSE60424, NK; and GSE60424, T cells [20]), were retrieved from the ADEx database (https://adex.genyo.es/ accessed on 3 November 2022). The SWISS-MODEL-modeled PABPC1 structure orientation resembles that generated by the Alpha Fold tool [21] (retrieved from Uniport; https://www.uniprot.org/ accessed on 3 May 2021). Superimposing both structures (SWISS-MODEL [16,17]) and “Alpha Fold” tool [21]), and tracing stability of individual residues were performed in the MOE (Chemical Computing Group Inc., Montreal, QC, Canada) package.

Frequently occurring mutations for the PABPC1 protein in different cancer types were investigated for the stability change of the protein. Individual variants were inserted in the PABPC1 structure, along with the predicted change in the stability (∆Stability or dStability, kcal/mol) using the “residue scan” module from the MOE (Chemical Computing Group Inc., Montreal, QC, Canada) package. During implementing the “residue scan” over the PABPC1 protein, the “LowModeMD” ensemble was used, and the CHARMM27 forcefield was applied [22]. In addition, the following parameters were set: 10,000 K search conformations and 50 rounds of iterations. The dStability for a particular mutation is the relative thermostability of the wild-type residue (Boltzmann average).

The PABPC1 protein in apo-form (crystal structure, pdb id: 4f02 [2]; the modeled system) as well as poly(A) mRNA were investigated further using the molecular dynamics simulation (MDS) technique. MD simulations on each system were performed using the GROMACS 4.6.5 (GROningen MAchine for Chemical Simulations) package [23] and applying the CHARMM27 forcefield. Individual systems were solvated in water (simple point charge model; SPC) containing N^+^Cl^-^-neutralizing ions, and in a 10 Å-thick dodecahedron simulation box. In this dodecahedron box, the periodic boundary conditions (PBCs) were implemented and systems with the solvent molecules were energy-minimized using the steepest descent algorithm. The Particle Mesh Ewald (PME) method [24] and the LINCS algorithm [25] were used to maintain electrostatic interactions (van der Waals and Coulomb interaction cut-off distance was set to 10 Å) and constrain bond lengths, respectively. After equilibrating systems for 1000 ps (in NPT isobaric–isothermal ensemble simulation), the production run was performed for 100 ns using the leapfrog integrator [26]. The V-rescale thermostat [25] was used to maintain the temperature of each system at 300 K. In addition, the Parrinello–Rahman barostat [23] was used to maintain the pressure at 1 bar. The PABPC1-poly(A) mRNA hydrogen bonding interactions were computed using the GROMACS and VMD tools [15], maintaining the donor–acceptor distance at 3.5 Å and angle cut-off at ≥160°–180°. Protein structure details retrieved from the molecular dynamics or molecular modeling were visualized using the VMD [15], MOE (Chemical Computing Group Inc., Montreal, QC, Canada), and BIOVIA Discovery Studio (Dassault Systèmes, BIOVIA Corp., San Diego, CA, USA) packages.

## 3. Results and Discussion

**Figure 2:**
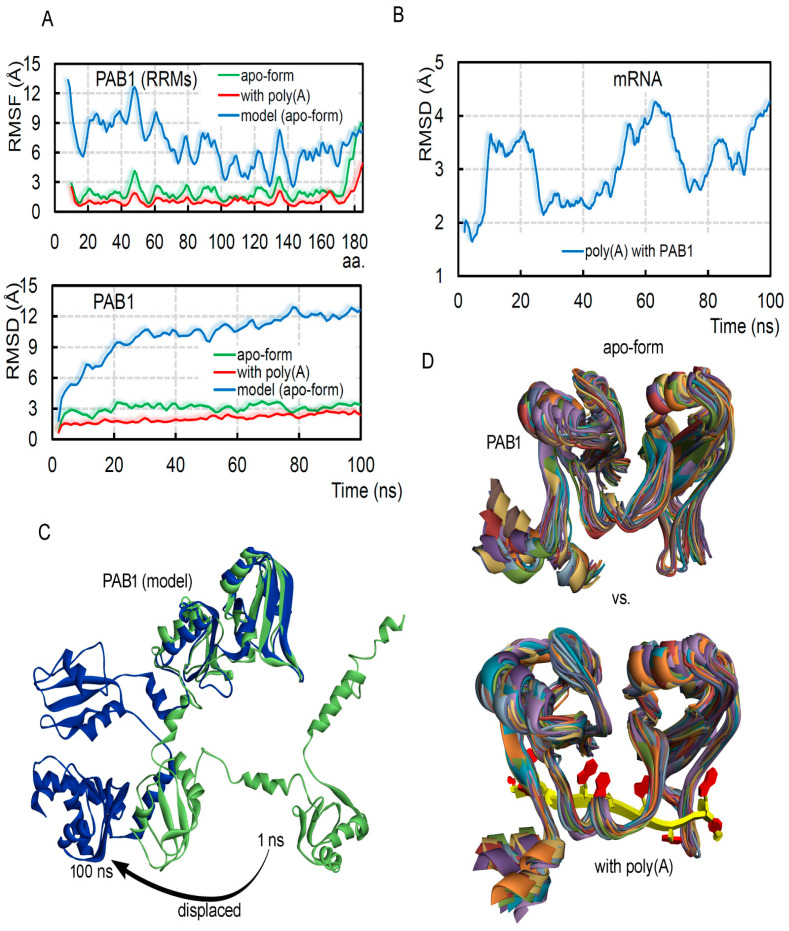
Dynamics of the PABPC1 or PAB1 protein, influenced by the presence of poly(A) mRNA. (**A**) RMSFs (root-mean-square fluctuation) of individual amino acids of RRM1–2 domains from PAB1 in different conditions, as well as the RMSDs (root-mean-square deviation, excluding hydrogen atoms) of the PAB1 structure. (**B**) The RMSDs for the poly(A) mRNA motif (5-′AAAAAAAAA-3′) in the system with the PAB1 protein. (**C**) The conformational dynamics or the folding of the modeled PABPC1 structure (RRM1–4) observed during 100 ns MD simulations, superimposed with the simulated crystal structure (RRM1–2). (**D**) The protein coordinates retrieved from different time frames of the MD simulations for the RRM1–2 domains from PAB1 complexed with poly(A) mRNA or apo-form.

**Figure 3:**
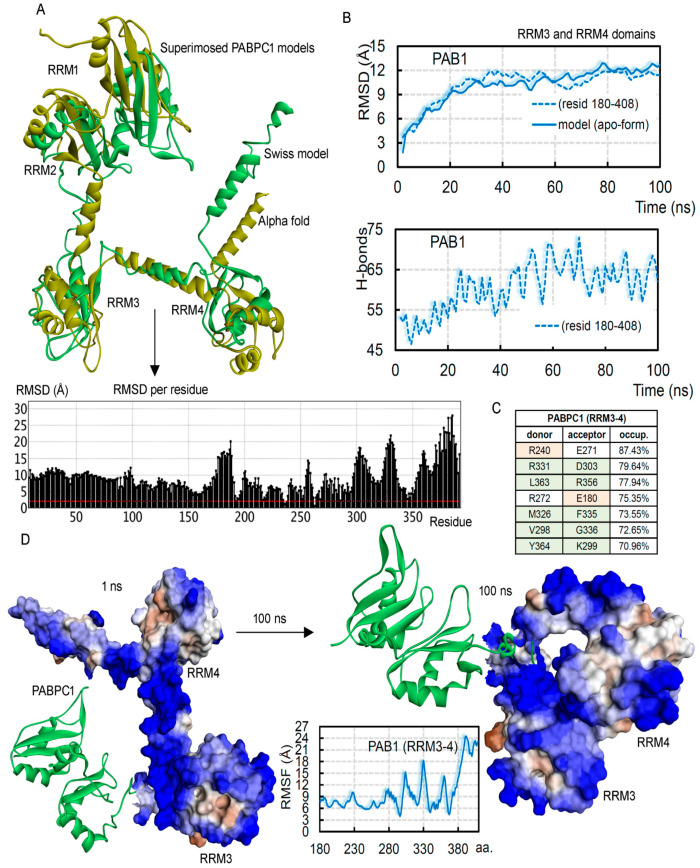
The RRM3–4 domains from the PAB1 and PABPC1 protein. (**A**) Our studied model of PABPC1 structure (generated using SWISS-MODEL [16,17]) superimposed with the structure generated by the “Alpha Fold” tool [21]. The below graph represents the RMSDs of individual residues when compared with both models. Structures were superimposed using the MOE (Chemical Computing Group Inc., Montreal, QC, Canada) pipeline. (**B**) The RMSDs of RRM3–4 domains from the molecular dynamics simulation were computed from the simulated PABPC1 apo-form structure. The below graph represents intramolecular interactions within the PABPC1 apo-form for the region 180–408 aa consisting of the RRM3–4 domains. (**C**) High-occupancy intramolecular interactions from the PABPC1 protein reside in RRM3 (orange background) and RRM4 (green background). (**D**) Conformational change in the PABPC1 apo-form and the hydrophobicity (blue for hydrophilic and brown for hydrophobic) also showed significant fluctuations. The center plot represents the RMSFs for the residue in the RRM3–4 domains.

In cancer cells, high expression of the PABPC1 protein can induce binding with different proteins (e.g., eIF4G; Figure 1B), and several such binding partners were elucidated by the cryo-EM or crystal studies [1,2,14]. However, the dynamics of full-length human PAB1 is the direction that needed further investigation, and therefore, using homology modeling along with molecular dynamics techniques, we measured structural properties of this gene (Figure 2). The crystal structure of the PAB1 protein consists of only two RNA recognition motifs (RRMs; RRM1–2 domains) with poly(A) mRNA (pdb id: 4f02 [2]). To implement the homology modeling, we modeled the possible full-length structure of all four RNA recognition motifs (RRM1–4; Figure 1C and Figure 2). The modeled PAB1-mRNA and apo systems were optimized or energy-minimized by applying the CHARMM27 forcefield in the MOE package (Chemical Computing Group Inc., Montreal, QC, Canada) [22]. The “Ramachandran plot” demonstrated in Figure 1C highlights the well-defined secondary structures that were modeled for the protein. Moreover, this modeled PABPC1 structure orientation resembled that generated by the “Alpha Fold” tool [21] (retrieved from Uniport; www.uniprot.org accessed on 3 May 2021). Superimposing both structures (SWISS-MODEL [16,17]) and “Alpha Fold” tool [21]) showed that when the RRM4 domain was excluded, the majority of the structure had less flexibility (Figure 3). These structures were further investigated using the MD simulation approach, along with considering different systems with the poly(A) mRNA.

Measuring the stability based on the RMSFs (root-mean-square fluctuation) or RMSDs (root-mean-square deviation, excluding hydrogen atoms) for the PABPC1 structure demonstrated that the presence of poly(A) mRNA induced stability within the residues (Figure 2A,B). In addition, the modeled PAB1 structure (8–408 aa; RRM1-4 domains) obtained a pattern of fluctuations similar to that of other simulated crystal structures consisting of RRM1–2 domains (10–184 aa; pdb id.: 4f02 [2]; Figure 2A and Videos S1–S2). In particular, the poly(A) mRNA was found to be changing its conformations, which stabilized by the end of the MD simulation time (Figure 2B). For the modeled apo-form of PAB1 (Model S1 and Video S3), the R176-Y408 residues formed a folded structure after a large displacement, as shown in Figure 2C and Video S3 (or Model S1), whereas the RRM1–2 domains were found to be conserved and less flexible, almost positioned like those in the crystal structure [2]. In comparing different conformations from MD simulation for PAB1 in the presence or absence of the mRNA motif, it was observed that the region binding with the poly(A) mRNA was more stable (Figure 2D) and gained a slightly different conformation compared to the apo-form.

Visualizing significant conformational folding in the RRM3–4 domains, we further investigated changes in the structural properties of these domains (RRM1–4; Figure 3). Extracting RMSDs from only the RRM3–4 domains (Figure 3B), it was observed that the majority of the protein flexibility emerged from these two RRMs within the PABPC1 structure. In addition, the intramolecular interactions among the RRM3–4 (180–408 aa) domains significantly increased over time (Figure 3B). RRM4 domain residues were mainly involved in high-occupancy intramolecular interactions (Figure 3C), despite their high flexibility (Figure 3D). In addition to conformational change, the surface hydrophobicity (mostly being hydrophilic) showed fluctuations (Figure 3D) in the PABPC1 apo-form.

**Figure 4:**
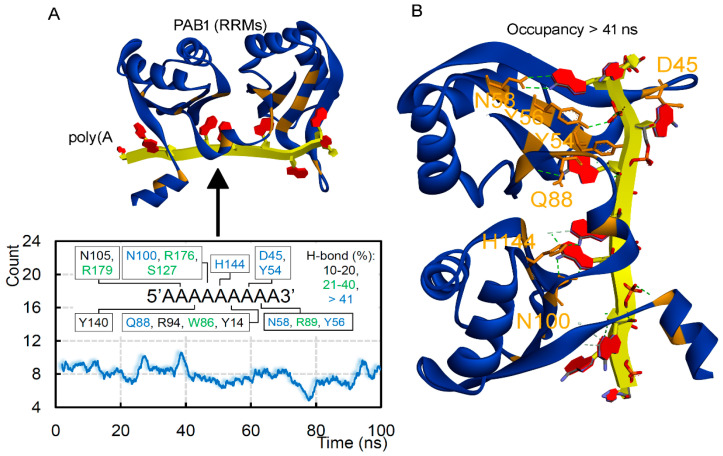
The binding patterns of PABPC1 with the poly(A) mRNA. (**A**) Intermolecular hydrogen bond interactions between PAB1-poly(A) mRNA, as well as individual amino acids interacting with nucleotides having an occupancy of ≥10% or nanoseconds (ns), are highlighted. (**B**) Position of high-occupancy (>41%) residues and their binding pattern with the poly(A) mRNA, represented over the PABPC1 structure.

The intermolecular interactions between poly(A) mRNA-PABPC1 suggest that ~8 H-bonds were formed every nanosecond (ns), and the PAB1 amino acids were found to be distributed over the mRNA (Figure 4A). The PAB1 residues D45, Y54, Y56, N58, Q88, and N100 formed long-lasting interactions having an occupancy ≥40% with the mRNA (Figure 4B). In particular, the poly(A) mRNA showed a unique pattern in the presence of PAB1. Every second nucleotide from both 5′ and 3′ ends was in an “inward-position” facing towards PAB1, whereas every third nucleotide had an “outward-position” conformation. The centered three nucleotides were found facing towards the region between RRM1–2 domains of the PABPC1 protein (Figure 2A,B). Specifically, aromatic amino acids from the PABPC1 protein (Y14, Y54, Y56, W86, and Y140) were found binding with poly(A) mRNA. These findings correlate with previously reported R94 and R179 residues involved in hydrogen bond interaction with the mRNA [9].

**Figure 5:**
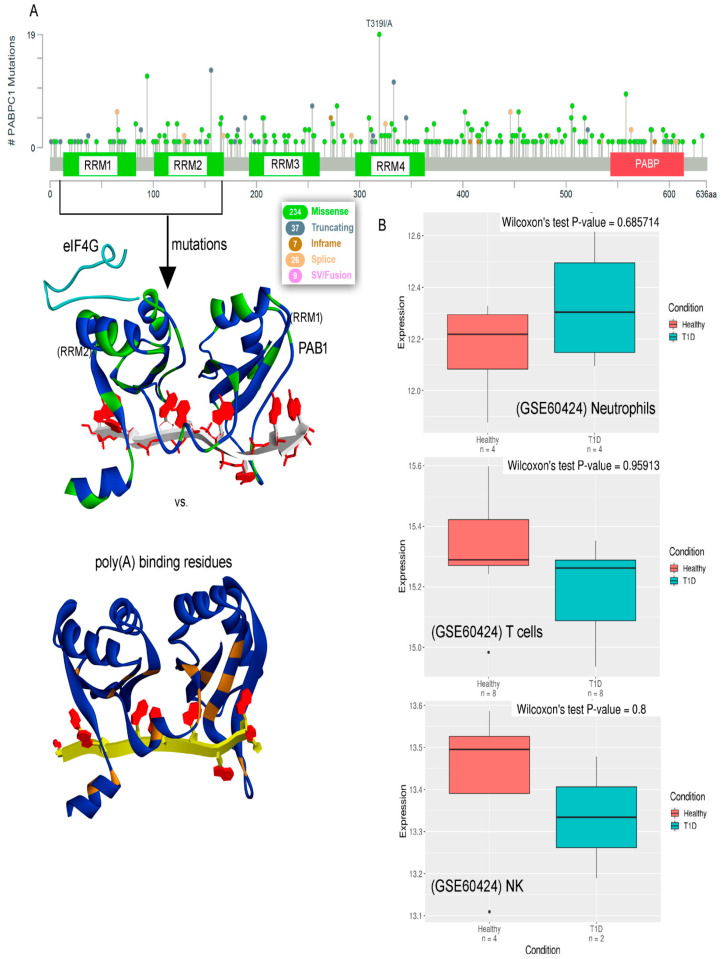
Cancer-derived mutations in the PABPC1 gene. (**A**) The mutation frequency of individual amino acids from different cancer types. Data retrieved from cBioPortal [19]. Residues involved in binding with the poly(A) mRNA from PAB1 were found to be preserved in different cancer types. (**B**) Boxplots representing expression profile of PABPC1 gene within a single dataset (type 1 diabetes (T1D) autoimmune disease; https://adex.genyo.es accessed on 3 November 2022), compared with the control samples (GSE60424, Neutrophils; GSE60424, NK; and GSE60424, T cells [20]).

PABPC1 has been shown to perform crucial roles in cancer and autoimmune diseases (ADs). Particularly, induced neoantigen production may lead to immune cell infiltration in the ADs, whereas in cancer cells, there is a lack of immune cell activation [11,27]. Deletion of PABPC1 protein in cancer cells could significantly increase the production of novel peptide read-through [11,12,13]. Moreover, the weighted gene co-expression network analysis (WGCNA) over microarray samples collected from ischemic stroke (IS) females revealed the expression profile of different genes, including the PABPC1 protein [27]. It has been proposed that in IS patients, a set of genes are linked to T cells, macrophages, NK cells, and neutrophils. In the samples studied by Haipeng et al. [27], it was demonstrated that the primary immune-infiltrating cells were neutrophils, resting NK cells, macrophages, and CD8 T cells. In particular, NK cells (large granular lymphocytes needed for IS immunosurveillance) blocking CD8  T cell activation were found regulating cellular immune response. Considering such involvement of PABPC1 in cell infiltration, we retrieved protein expression data for autoimmune diseases from the ADEx database (Autoimmune Diseases Explorer; https://adex.genyo.es/ accessed on 3 November 2022) [20]. This database covers common organ-specific autoimmune diseases (from >5000 samples), type 1 diabetes (T1D), rheumatoid arthritis (RA), etc. In particular, the expression profiles of PABPC1 in T1D from (GSE60424; Neutrophils, GSE60424; NK, and GSE60424, T cells [20]) different datasets are presented in Figure 5.

**Figure 6:**
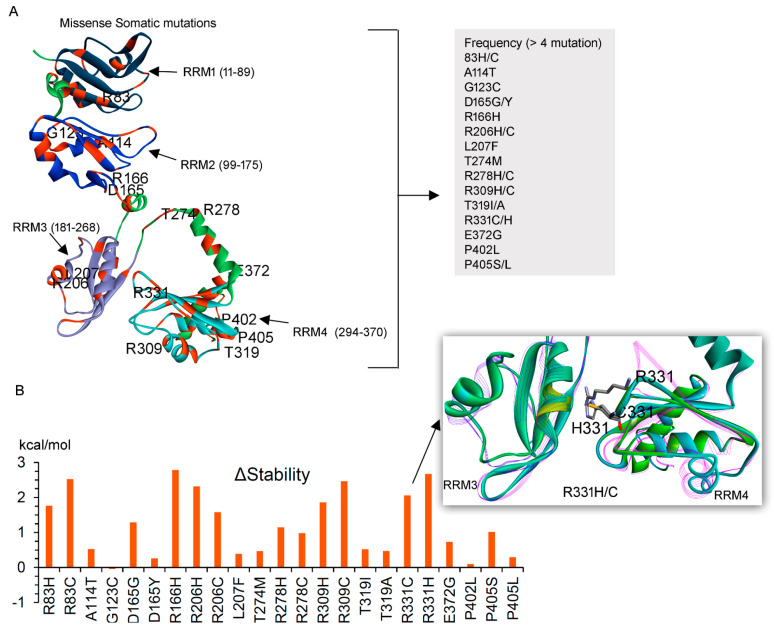
Influence of cancer-derived mutations over the PABPC1 structure. (**A**) Individual mutations from different cancer types (cBioPortal database [19]) represented over the PABPC1. Mutations with high frequency (>4; right panel) were further investigated via tracing stability changes (dStability) in the protein. RRMs are labeled according to their location: RRM1 (11–89 aa), RRM2 (99–175 aa), RRM3 (181–268 aa), and RRM4 (294–370 aa). (**B**) Change in stability for PABPC1 structure upon inserting point mutations. Residue scan module implemented in the MOE (Chemical Computing Group Inc., Montreal, QC, Canada) was implemented to trace the dStability (kcal/mol). The right panel represents the R331H/C mutations significantly reducing the protein stability.

Moreover, the dataset describing or containing mutation frequency of individual amino acids from different cancer types was retrieved from the cBioPortal (Figure 5A) [19]. The majority of the PABPC1 point mutations in cancer reside within the RRM domains, and in particular, the regions within 300350 aa (RRM4 domain), have high mutation frequency (Figure 5A). Comparing these data with the hydrogen-binding residues of the poly(A) mRNA, it was observed that amino acids engaging in long-lasting interactions were found to be preserved in different cancer types (Figure 5A); i.e., none of them were found to be mutated in the retrieved cancer dataset. Furthermore, mutations with high frequency (>4) were investigated to trace changes in the stability of the protein structure (Figure 6). However, in the G123C variant, the majority of the cancer-derived mutants reduced the stability of the protein (Figure 6B). In particular, both R331H and R331C mutations (Figure 6B) significantly disrupted the PABPC1 structure.

**Figure 7:**
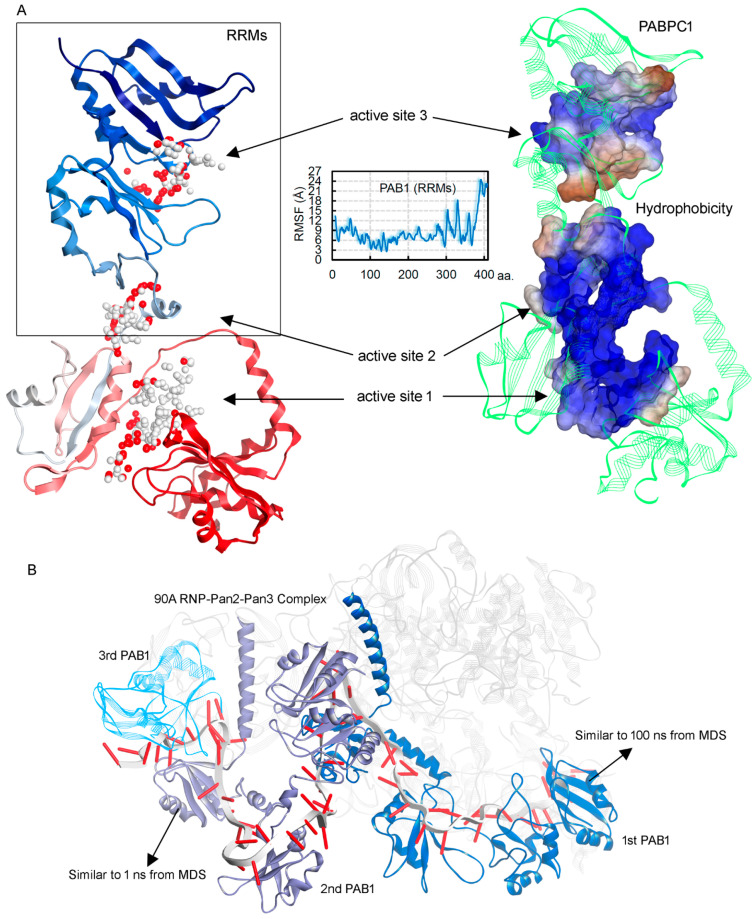
Predicted active sites over the modeled PABPC1 structure that was retrieved at the end of MD simulation, using the “Alpha Shapes” construction geometric method [18]. (**A**) Three large sites were found using this “Alpha Shapes” approach. The center RMSF plot represents the RRM3–4 domain residues having high flexibility due to conformational switch within the protein structure. The right panel describes the hydrophobicity (blue for hydrophilic and brown for hydrophobic) of the active sites. (**B**) The cryo-EM structure describing multiple binding conformations of the PABPC1 protein in the 90A RNP-Pan2-Pan3 complex (pdb id.: 6r5k [14]). These conformations resemble our simulated PAB1 model, i.e., from the beginning and end of MD simulations.

In addition, one of the active sites (from three active sites built using the “Alpha Shapes” approach) originated in the region (300–350 aa; RRM4 domain) that is highly mutated in different cancer types (Figure 7A and Table 1). For most of the eukaryotic mRNAs, stability depends on a ribonucleoprotein, and PAB1 is known to be bound with the poly(A) tail [14]. This PAB1 protein at the poly(A) tail can bind with different proteins, gaining slight structural conformations or movements. Cryo-EM data have shown multiple binding conformations or structural folding of the PABPC1 protein in the 90A RNP-Pan2-Pan3 complex [14]. Different structural movements observed in our MD simulations can be directly correlated with such dynamics traced in the cryo-EM studies [14]. Our initial conformation from beginning of MD simulation was almost identical to the second PAB1 (as shown in Figure 7B), and structure from end of MD simulation correlates with the first PAB1 (Figure 7B) molecule. Moreover, it is known that in eukaryotic cells, diverse stresses can enhance coalescence of RNA-binding proteins, making them stress granules. Similarly, the PABPC1 protein has been suggested as a defined marker of stress granules under physiological stress conditions [28]. Since multiple conformations were observed in our MD simulations, it could be hypothesized that in cellular stress, PABPC1 could represent distinct folding, affecting the predicted active sites (Figure 7A and Table 1). However, we believe that in non-stress conditions, PABPC1 may form the active sites presented in Figure 7.

## 4. Conclusions

The PAB1 or PABPC1 protein is proposed to induce mRNA stability; hence, it is suggested as an antagonist of the NMD factors when targeting mRNA Despite this, it has been found that deletion of either PAB1 or UPF1 (NMD) significantly increases the production of novel peptide read-through. This could result in the increased production of mutant peptides that can be presented over the HLA molecules and could trigger the immune response in cancer. Hence, applying different target inhibition strategies to completely or partially block the activity of PAB1 may result in induced production of peptide read-through over mRNA, which eventually produces mutant peptide or neoantigens in cancer cells. A detailed understanding of the PAB1 protein structure can guide such experimental designs to change the activity of this protein. Herein, we investigated the structural folding or dynamics (correlating with the cryo-EM) of the human PAB1 protein, and proposed several key residues involved in mRNA binding, as well as highlighted different active sites.

Applying the homology modeling techniques, we modeled and optimized the possible full-length structure of all four RRMs for the PAB1 protein. For this PAB1 model, it has been observed that the residue range R176-Y408 initiated a folded structure after a large displacement. The D45, Y54, Y56, N58, Q88, and N100 residues formed long-lasting high-occupancy interactions with the poly(A) mRNA. Monitoring the specific mRNA selectivity for PAB1, we reviewed aromatic amino acids (Y or W) associated extensively with the poly(A) mRNA. On the other hand, the poly(A) mRNA has shown a unique pattern towards the PAB1 protein. Every second nucleotide from both the 5′ or 3′ ends was in an “inward-position” facing towards protein, whereas every third nucleotide had an “outward-position” conformation, and the center three nucleotides faced towards the region between RRM1–2 domains. Moreover, residues from the RRM4 domain were mainly involved in high-occupancy intramolecular interactions, despite high flexibility within its residue. The majority of the high-frequency cancer mutations in PAB1 reside within the RRM domains, and in particular, the regions within 300–350 aa (RRM4) have high mutation frequency. Amino acids engaging in long-lasting interactions with poly(A) mRNA were found to be preserved in different cancer types. However, in the G123C variant, the majority of the cancer derived mutants reduced the stability of the protein. We believe that the molecular details from this study provide a detailed understanding of the PABPC1 structure, and can guide future in vitro or in vivo experiments to modulate the activity of this gene.

## Figures and Tables

**Table 1:** Active site predicted over the PABPC1 modeled structure using the “Alpha Shapes” construction geometric method [18] implemented in the MOE (Chemical Computing Group Inc., Montreal, QC, Canada) package.

Active Sites	Residues
No. 1	R176, K177, E180, H241, E242, Q245, K246, V248, D249, N252, G253, G264, R265, A266, Q267, K268, R272, Q273, T274, K299, N300, L301, D302, D303, E328, R331, S332, K333, G334, F335, T360, K361, P362
No. 2	N105, L162, D165, R166, K167, F169, S175, R176, K177, E178, E180, A181, G184, A185, R186, A187, K188, E189, F190, E239, R240, E242, D243, K246, R272
No. 3	Y14, G16, D17, L18, H19, P20, L52, G53, Y54, N73, F74, D75, G79, K80, P81, V82, R83, I110, D111, N112, K113, K129, V130, V131, C132, D133, F142

## Data Availability

Data is contained within the article or supplementary material.

## Supplementary Materials

The following supporting information can be downloaded at: https://www.mdpi.com/article/10.3390/biomedicines10112981/s1, Model S1. The tertiary structure coordinates for the modeled PABPC1 protein retrieved at the end of the MD simulations (100 ns). Video S1. Conformational changes in the PABPC1 protein in the apo-form over the MD simulation time. Video S2. Conformational changes in the PABPC1-poly(A) mRNA complex over the MD simulation time. Video S3. The conformational dynamics of the modeled PABPC1 structure observed during 100 ns MD simulations.
